# Clinical study of apatinib combined with EGFR‐TKI in the treatment of chronic progression after EGFR‐TKI treatment in non‐small cell lung cancer (ChiCTR1800019185)

**DOI:** 10.1111/1759-7714.13303

**Published:** 2020-01-09

**Authors:** Xin Li, Minghui Liu, Hongbing Zhang, Hongyu Liu, Jun Chen

**Affiliations:** ^1^ Department of Lung Cancer Surgery Tianjin Lung Cancer Institute, Tianjin Medical University General Hospital Tianjin China; ^2^ Tianjin Key Laboratory of Lung Cancer Metastasis and Tumor Microenvironment Tianjin Lung Cancer Institute, Tianjin Medical University General Hospital Tianjin China

**Keywords:** Apatinib, EGFR‐TKIs, non‐small cell lung cancer, slow progression

## Abstract

This clinical trial (ChiCTR1800019185) is designed to be an open‐label, prospective, single‐center, single arm exploratory research study. The study will recruit non‐small cell lung cancer patients (NSCLC) with slow progression after first‐line treatment with EGFR‐TKI drugs. Slow progression will be confirmed by the presence of serum carcinoembryonic antigen or imaging evaluation. The primary aim is to assess progression‐free survival after EGFR‐TKIs treatment combined with apatinib 250 mg once daily. The secondary objectives are to evaluate objective efficacy, disease control rates, quality of life, overall survival, and safety. From September 2018 to September 2020, under specific entry and discharge standards, we plan to enroll 38 eligible patients until the end of the study. We hope that our study will help to explore a new way of combining the small molecular inhibitors of antiangiogenesis with EGFR‐TKIs to overcome acquired drug resistance.

## Introduction

Lung cancer is the most common malignant tumor worldwide. Non‐small cell lung cancer (NSCLC) accounts for 80% of lung cancers.^1^ In Chinese males, lung cancer is the most common tumor type and has the highest mortality rates. In Chinese females, lung cancer is the second most common tumor type, and has the highest mortality rates. According to the United States Surveillance, Epidemiology, and End Results database, 57% of lung cancer patients have distant metastases at the time of initial diagnosis.^2^ Therefore, the treatment of advanced patients is an important part of lung cancer treatment. Nonsquamous cell NSCLC accounts for approximately 70% of NSCLC. If not treated promptly, it will spread to other organs of the body, causing serious complications. EGFR‐TKI has been approved for the first‐line treatment of advanced NSCLC patients with EGFR‐positive mutations.^3^ However, some patients develop drug resistance after using EGFR‐TKI drugs. In most cases, this is due to a point mutation in position 790 of EGFR 20 exon, one of the most recognized drug resistance mechanisms.^4^ There are two manifestations of drug resistance in clinical practice, slow progression and explosive progression. According to the response evaluation criteria in solid tumors (RECIST) 1.1 tumor staging criteria, the clinical manifestation of patients with slow progression is between stable disease (SD) and progressive disease (PD). Tumor diameter and relative increases of 0%–20% is one of the criteria for slow progression. In slow progression NSCLC, there is no clear standard of care, and EGFR‐TKI can be considered in combination with antiangiogenic drugs.

Angiogenesis is a key step in the development and progression of malignant tumors.^5^ It is generally believed that angiogenesis in the tumor area provides nutrients and entraps metabolites, allowing tumor cells to be transferred to other parts of the body through new blood vessels. Therefore, effective inhibition of angiogenesis in tumor areas can inhibit the growth of tumor cells while reducing the occurrence of metastasis. At present, antitumor angiogenesis has become a promising new strategy for cancer treatment. The development of tumor neovascularization is associated with a variety of vascular‐related factors. Rapidly growing tumor cells can secrete multiple vascular growth factors under hypoxia, stimulating tumor angiogenesis. One of the most important growth factors is vascular endothelial growth factor (VEGF). VEGF binds to the VEGF receptor (VEGFR), stimulates VEGFR‐mediated downstream signaling, and ultimately leads to tumor angiogenesis.^6^ It is thought that VEGFR and human epidermal growth factor receptor 1 (HER‐1)/epidermal growth factor receptor (EGFR) share a common downstream signaling pathway, so EGFR‐TKI can also downregulate VEGF. Studies have shown that VEGF inhibitors can also inhibit HER‐1/EGFR signaling. VEGFR‐2 is a major mediator of endothelial cell function in VEGF. When endothelial cells are stimulated by VEGF, VEGFR‐2 promotes cell proliferation mainly through the PKC/RAF/MAP pathway and modifies or activates various signaling molecules. These include Akt, ERK‐2, PI3K, FAK, and Caspase‐9. Activation of Akt/PI3K can promote the downstream signaling cascade of EGFR, and there is cross‐reactivity between the two pathways. Theoretically, the combination of the two has at least an additive effect.

Carcinoembryonic antigen (CEA) is an acidic glycoprotein with the characteristics of human embryonic antigen. It exists on the surface of cancer cells differentiated from endoderm cells and is the most widely used broadspectrum tumor marker in clinical practice. CEA is present in 40%–80% of lung cancers, and is associated with tumor metastasis and recurrence. As such, it is of prognostic significance.^7^ Dynamic monitoring of CEA levels to reflect efficacy has been recognized. Multiple studies have found that CEA levels in PD patients are higher than before treatment. As such, we will use imaging to determine the sum of the diameters of all measured target lesions throughout the experiment, and this will be used as a reference point. Screening criteria for enrolling patients will include RECIST tumor staging criteria, screening patients with tumor diameters and relative increases of 0%–20% after previous EGFR‐TKI monotherapy, and increased CEA levels after previous EGFR‐TKI monotherapy.^8^

Previous studies have shown that EGFR‐TKI combined with an antiangiogenic drug (bevacizumab) is effective in the treatment of advanced NSCLC, and can improve patient survival.^9^, ^10^, ^11^, ^12^ The clinical study of bevacizumab combined with erlotinib in EGFR‐TKI resistance has also made breakthroughs. In the BELIEF study published at the 2015 European Cancer Congress, patients with advanced nonsquamous NSCLC and EGFR exon 19 deletion or exon 21 (L858R) mutation were enrolled. The study investigated the effects of erlotinib plus bevacizumab treatment on PFS in patients with or without EGFR T790M mutation.^13^ Preliminary results indicated that all patients, T790M‐positive patients, and T790M‐negative patients showed progression‐free survival (PFS) of 13.8, 16.0, and 10.5 months, respectively, which appeared to indicate that antiangiogenic drugs plus EGFR‐TKI were suitable for patients with T790M mutations. The results need to be further explored.

Apatinib is a small molecule VEGFR tyrosine kinase inhibitor (TKI) developed by Hengrui Pharmaceutical Co., Ltd. with independent intellectual property rights. The drug mainly inhibits VEGFR‐2 to exert antiangiogenic effects to treat malignant tumors. in vitro and in vivo tests have shown that apatinib has good antitumor activity against lung cancer. In a phase 2 randomized, double‐blind, placebo‐controlled trial, this product was observed as a third‐line treatment for patients with advanced nonsquamous NSCLC. Compared with the placebo group, median progression‐free survival (mPFS) was prolonged by 2.8 months.^14^ Phase 3 clinical trials are still ongoing.

In summary, angiogenesis inhibitors combined with EGFR‐TKI can significantly improve the efficacy of EGFR‐TKI in EGFR‐positive patients, delaying the occurrence of drug resistance. T790M‐positive disease is the main indication for TKI treatment of NSCLC resistance, followed by slow progression. Ideal initial data would suggest that antiangiogenic drugs plus EGFR‐TKI treatment of T790M‐positive patients have certain clinical significance.

**Figure 1:**
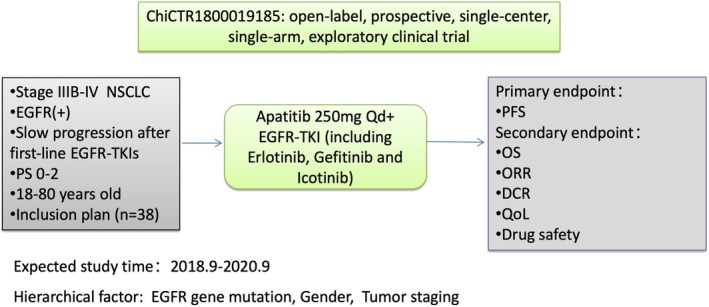
The study design flow.

This study will investigate the efficacy and safety of continuous EGFR‐TKI and apatinib (angiogenesis inhibitor) treatment in patients with NSCLC determined to have slow progression by CEA or imaging after first‐line treatment with EGFR‐TKI (including erlotinib, gefitinib, and icotinib). In addition, the study will analyze the correlation between clinical characteristics (EGFR gene mutation status, gender, staging, etc) and therapeutic efficacy. The study design flow is shown in Figure 1.

### Primary endpoint

PFS: the time from randomization to patients experiencing tumor progression or death.

### Secondary endpoints

1. Overall survival (OS): time from randomization to death from any cause.
2. Objective response rate (ORR): refers to the proportion of patients whose tumor shrinkage has reached a certain level and maintained for a certain period of time, including cases of complete response (CR) and partial response (PR). RECIST 1.1 criteria were used to evaluate objective tumor remission. The patients must have measurable tumor lesions at baseline. According to RECIST 1.1, the evaluation criteria of efficacy can be divided into CR, PR, SD and PD.
3. Disease control rate (DCR): refers to the percentage of patients with confirmed CR, PR and disease stability (≥4 weeks) in the patients with evaluable efficacy.
4. Quality of life score (QoL), refers to the EORTC QLQ‐C30 (version 3, Chinese version) evaluation method. The questionnaire evaluates changes in clinical symptoms and objective examination results of tumor patients before and after treatment. The results of each field of the QoL scale will be recorded in the electronic case report form (eCRF).
5. Drug safety: patients will be monitored for adverse events occurring during the period of clinical research. In addition, patients will undergo clinical laboratory tests, and clinical characteristics will be monitored, including severity, occurrence time, duration, treatment and prognosis. Any correlation between experimental drugs will be documented. Common Terminology Criteria for Adverse Events (CTCAE) version 4.0 will be used to categorize adverse events.

### Study design

#### Description of research design

This is an open‐label, prospective, single‐center, single‐arm, exploratory clinical trial to observe and evaluate the efficacy and safety of continued apatinib combined with EGFR‐TKI in NSCLC patients with slow progression after EGFR‐TKI monotherapy.

#### Overall study design

This research will be conducted in a single center, in the lung cancer surgery department of Tianjin Medical University General Hospital. Patients will be withdrawn from the study for any of the following reasons:

- Withdrawal of consent
- Drug toxicity or intolerance
- The principal investigators considers the patient is unfit to continue in the study.

During the study, patients will undergo imaging to monitor tumor progression. Efficacy and safety endpoints will be monitored throughout the study.

#### Experimental drugs

Name: Apatinib

Specification of preparation: 0.25 g (10 tablets/plate/box)

Preparation unit: Jiangsu Hengrui pharmaceutical co., Ltd

Validity period: 24 months

Route of administration: oral administration

Storage conditions: shading, sealing, below 25°C

#### Patient selection and inclusion criteria

Diagnostic criteria: the diagnosis will be confirmed according to the sixth edition of Oncology Diagnostics and the eighth edition of Internal Medicine published by the People's Medical Publishing House of the People's Republic of China.

#### Inclusion criteria

1. Males or females ˃18 years old and <80 years old.
2. Diagnosed with advanced stage (IIIB or IV), nonsquamous NSCLC, with measurable lesions (per RECIST 1.1 criteria).
3. NSCLC patients with slow progression determined by (i) disease control lasting ≥3 months with EGFR‐TKI treatment, (ii) compared with the previous assessment, no significant increment of tumor burden and progressive involvement of nontarget lesions with a score ≤2, or PD due to solitary extracranial lesion or limitation in intracranial lesions (covered by a radiation field), or the abnormal CEA level is increasing higher than pre‐EGFR‐TKIs treatment during the last three months with a score ≤2, and (iii) symptom scored ≤1.
4. There is no interventional systemic treatment between cessation of gefitinib or erlotinib and initiation of new treatment.
5. Eastern Cooperative Oncology Group (ECOG) performance status (PS) score: 0–2.
6. Expected survival ≥12 weeks.
7. No previous chemotherapy or other targeted therapy. For patients with chemotherapy as first‐line treatment and EGFR‐TKIs as second‐line treatment.
8. Patients may have been treated with radiation previously, but the radiation area must be less than 25% of the bone marrow area^15^ and radiation therapy must end at least four weeks before being enrolled into the group.
9. Main organs are functioning normally (ie, no blood transfusion or blood products within 14 days, no correction with granulocyte‐colony stimulating factor and other hematopoietic stimulators):
  - Blood routine test must be:
    - Hemoglobin ≥90 g/L
    - Absolute neutrophil count ≥1.5 × 10^9^/L
    - Platelets ≥80 × 10^9^/L or higher
  - Blood biochemical tests must be:
    - Total bilirubin <1.5 × upper limit of normal (ULN)
    - Alanine aminotransferase and aspartate aminotransferase <2.5 × ULN or <5 × ULN in patients with liver metastasis
    - Serum creatinine ≤1.25 × ULN or endogenous creatinine clearance >45 mL/minute
  - Females of reproductive age must have a negative pregnancy test (serum or urine) within seven days prior to enrollment and voluntarily use appropriate methods of contraception during observation and for eight weeks after the last apatinib tablet. Males must be surgically sterilized or voluntarily consent to use appropriate contraceptive methods during the observation period and for eight weeks after the last apatinib tablet is given.
  - Patients volunteered to participate in this study and signed the informed consent (ICF), with good compliance and follow‐up.

#### Exclusion criteria

1. Persistent clinical treatment‐related toxicity associated with previous chemotherapy and/or radiotherapy.
2. Receive radiotherapy (except for limbs and brain) within three months prior to baseline imaging examination.
3. Symptomatic brain metastases (patients with brain metastases who have completed treatment 21 days before enrollment and are confirmed to have no symptoms of cerebral hemorrhage and stable symptoms can be enrolled).
4. Radiological evidence suggests the presence of vacuous or necrotizing tumors.
5. Imaging shows that the distance of the primary tumor from the great blood vessel is ≤5 mm; or the primary tumor invaded the local great blood vessel; or have obvious lung cavity/necrotizing tumor.
6. Coagulant function abnormality (international normalized ratio > 1.5 × ULN, or activated partial thromboplastin time > 1.5 × ULN) with bleeding tendency, or is treated with thrombolysis and anticoagulation and antiplatelet drugs, including obvious hemoptysis/daily hemoptysis of two teaspoons or above within two months of enrollment, clinically significant bleeding or bleeding tendency within three months of enrollment, venous thromboembolism events within one year of enrollment, or known other inherited or acquired bleeding and thrombosis tendency.
7. Patients with hypertension who do not drop to the normal range after antihypertensive medication (systolic blood pressure > 140 mmHg, diastolic blood pressure > 90 mmHg).
8. Long‐term open wounds/fractures.
9. ˃level II or poorly controlled cardiovascular disease.
10. Squamous cell carcinoma (including adenosquamous cell carcinoma); small cell lung cancer (including mixed small cell and non‐small cell lung cancer).
11. Patients with a history of interstitial lung disease or who also have interstitial lung disease.
12. In the judgment of the principal investigators has any other conditions that might influence the outcome of the study.

#### Criteria for excluding patients from analysis

Study drugs were not taken in accordance with the protocol, making it impossible to evaluate efficacy and/or safety.

## Serious protocol violations

#### Patient withdrawal criteria

1. Patient withdraws informed consent
2. Medical imaging examination shows PD
3. Pregnancy during the trial
4. Principal investigator discretion

### Dosage regimen

Apatinib: 250 mg orally, once daily; EGFR‐TKI: dose determined by principal investigator.

### Clinical data collection

#### Screening period (14 days before treatment)

Once the patient signs the ICF, they will undergo the following evaluations: medical history collected, vital signs, physical examination and blood pressure, coagulation function, electrocardiogram (ECG), ECOG PS score, routine blood tests, routine urine tests, routine stool tests, liver and kidney function, electrolyte, tumor markers, biomarkers, CEA, imaging examination (computerized tomography [CT]/magnetic resonance imaging [MRI]).

### During treatment (every four weeks for a cycle, every two cycles for the efficacy evaluation)

#### Singular period

Patients will undergo the following evaluations: vital signs, physical examination, blood pressure, coagulation function, ECG, ECOG PS score, routine blood tests, routine urine tests, routine stool tests, liver and kidney function, electrolyte, tumor markers, biomarkers, CEA, QoL score, adverse events, and concomitant medication.

#### Even period

Patients will undergo the following evaluations: vital signs, physical examination, blood pressure, QoL score, adverse events, and concomitant medication.

#### After treatment (within 30 days after the end of treatment)

Patients will undergo the following evaluations: adverse events, concomitant medication and QoL.

## Safety parameters

### Adverse events observation

#### Adverse event definition

Adverse events are any adverse medical events that occur after a subject or clinical trial subject receives a drug or treatment regimen but are not necessarily cause‐and‐effect.

Adverse events may be any signs (including abnormal laboratory results), symptoms, or diseases that are unpleasant or unrelated to the purpose of the use of the product and that have a temporal association with the use of the product, whether or not they are considered to be related to the medical product.

According to the regulations, events that occur before and after treatment are also considered adverse events. Therefore, reports of safety monitoring adverse events or serious adverse events should begin when subjects enter the trial (after signing the ICF and passing screening) and continue until the final follow‐up visit.

#### Adverse event classification

Adverse events will be classified by CTCAE version 4.0, according to the National Cancer Institute (NCI) standard of classification for common acute and subacute toxicity. Adverse events not listed in the NCI toxicity classification criteria can be judged according to the following criteria:

- Degree 1 (mild): uncomfortable feeling, but does not affect normal daily activities
- Degree 2 (moderate): the level of discomfort is not enough to reduce or affect normal daily activities
- Degree 3 (severe): inability to work or normal daily activities
- Degree 4 (fatal): disabling or fatal

#### Recording adverse events

Details of adverse events during the trial, including severity, time, duration, and relation to study treatments will be recorded in the eCRF. Abnormal laboratory results will recorded in the eCRF, and will be followed up at least weekly until the abnormal result normalizes or the study is completed.

#### Judgment of the relationship between adverse events and study drugs

Relationship between adverse events and study drugs will be categorized as follows.

#### Related

In accordance with the known type of reaction of the drug used, in accordance with the reasonable time sequence after the use of the drug, reduce or stop the drug to reduce or stop the drug to decrease the adverse reaction, again after the administration of the adverse reaction.

#### May be related

Consistent with the known type of reaction to the drug used, consistent with a reasonable sequence of time after administration, reduced or reduced or disappeared after withdrawal of the drug, but the patient's clinical status or other reasons may also cause this reaction.

#### May be not related

It does not conform to the known type of reaction of the drug used, does not conform to the reasonable time sequence after the drug is administered, and the patient's clinical status or other reasons may also cause the reaction.

#### Not related

It does not conform to the known type of reaction of the drug used, does not conform to the reasonable time sequence after the use of the drug, the patient's clinical state or other reasons can also explain the reaction, and the reaction is reduced or disappeared after excluding clinical symptoms or other reasons.

#### Indeterminable

The relationship between drugs used and adverse reactions cannot be explained.

#### Serious adverse event

If any Serious Adverse Event (SAE) occurs during the trial, the investigator should immediately take appropriate measures to protect the patients and report the SAE to the principal investigator within 24 hours. The investigator should fill in the SAE report form, sign and date the report. SAEs events include: death or life‐threatening; cause or prolong hospitalization; cause permanent disability; cause cancer: cause deformity: drug overdose.

#### Observation and management of adverse events

The study center is equipped with first‐aid drugs, oxygen, blood pressure meter, electrocardiograph, defibrillator, ventilator, and a series of drugs and equipment needed for routine infusion. Patients will be given the study drugs under the supervision of a clinician, and their vital signs and other adverse reactions will be observed periodically until the end of the study. According to different conditions of adverse events and SAEs, appropriate treatment measures will be taken for patients, and necessary rescue measures will be adopted in case of emergencies.

#### Management of adverse reactions

Generally, after the occurrence of minor adverse reactions, the clinician should first observe the condition, make a clear diagnosis and determine the prognosis. If the study is not affected, observation and symptomatic treatment and other measures should be taken to try to improve, cure or stabilize the reaction.

## Statistical methods

### Data management

The CRF should be completed by the principal investigator for each patient, and checked to ensure complete and accurate. After reviewing and confirming that the data is correct, key researchers, sponsors and statistical analysts will lock the database. After this point, no changes will be made to the locked data file.

### Statistical analysis of data

#### Data sets

There will be three sets of analyses in this study as follows.

#### Full analysis set

This will be comprised of all patients who receive at least one dose of the study drug. Data from the last observation carried forward (LOCF) will be used in cases where the full course of treatment was not observed.

#### Per protocol set

This will be comprised of all patients who conform to the study protocol, have good compliance, do not take prohibited drugs during the study, and have complete eCRFs. There will be no input of missing data in this data set.

#### Safety analysis set

This will be comprised of all patients who receive at least one dose of study drug and have any safety information recorded. This data set will be used for security analysis.

### Statistical analysis plan

All statistical analysis will be performed by SPSS 19 statistical analysis software. All statistical tests will be performed by bilateral test. *P*‐values ≤0.05 will be considered statistically significant, and the 95% confidence interval reliable.

Baseline data will be analyzed according to the full analysis set, and all efficacy indicators will be analyzed according to the full analysis set and the per protocol set. Security analysis will be performed using the safety analysis set.

Abscission analysis: descriptive analysis will be used to compare of total abscission rate. Abscission rate due to adverse events will be performed using a chi‐square test or Fisher's exact probability test.

Efficacy analysis: for indicators PFS and OS, the product limit method will be adopted and 25%, 50% (median), 75% PFS and OS at different times after treatment will be calculated, respectively according to the actual situation of the data.

Safety analysis: descriptive statistics will be used to describe the adverse events in this study. Laboratory test results will be summarized using descriptive statistics.

### Ethical considerations

An ethics committee and informed consent are the main measures to protect the rights and interests of patients. Before the clinical trial begins, the protocol will be considered and approved by the ethics committee of Tianjin Medical University General Hospital, and the approval opinions shall be signed before implementation. During the study, any modifications to the protocol will be approved by the ethics committee before implementation.

The principal investigators must explain to the patients that participation in the study is voluntary, that he/she has the right to withdraw from the study at any time and at any stage of the study without discrimination or retaliation, that his/her medical treatment and rights and interests are not affected, and that he/she can continue to receive other treatments. Patients will be made aware that their personal information used in the study will be treated confidentially. They will be informed of the purpose of the study, the expected benefits, risks and inconvenience. They will be informed of other available treatment options. The study will meet the requirements of the Declaration of Helsinki with regards to rights and obligations of patients. Patients will have sufficient time to consider whether or not they wish to participate in the study trials and will sign the ICF.

The protocol has now been approved by the ethics committee of Tianjin Medical University General Hospital (IRB2018‐124‐01) and also registered on http://www.clinicaltrials.org with No.ChiCTR1800019185.

## Discussion

The primary objective of this study is to investigate the efficacy of EGFR‐TKI combined with an angiogenesis inhibitor (apatinib) in patients with slow progression of NSCLC previously treated with EGFR‐TKI monotherapy (including erlotinib, gefitinib, and icotinib). Secondary objectives include monitoring safety, and further analysis of clinical characteristics (EGFR gene mutation status, gender, staging, past treatment, etc) and treatment efficacy.

The advantage of this prospective study is that apatinib is a small molecule VEGFR TKI with independent intellectual property rights in China. In a previous phase 2 trial, apatinib as a third‐line treatment for advanced nonsquamous NSCLC prolonged mPFS compared with placebo. Compared with other antitumor drugs with antiangiogenic effects, it has a good safety profile, and the method of administration is simpler and more convenient, with no hospitalization required. In addition, the treatment costs are lower.

However, attention should be paid to the potential limitations, including whether apatinib will be effective for combined use with EGFR‐TKIs in NSCLC patients. Whilst there is some previous relevant experimental data supporting their combined use, close attention will be paid to tumor progression, and if clear tumor progression occurs, patients will be withdrawn in a timely manner and treated according to Chinese Lung Cancer Treatment Guidelines.

In addition, in the exclusion criteria of this trial, we did not exclude the NSCLC patients with slow progression who had EGFR exon20 T790M by liquid NGS detection analysis. For those patients with EGFR T790M, they should benefit from the third generation TKIs such as Osimertinib, and these patients should be recommended to use third generation TKIs. We did not exclude these patients when we designed the trial because third generation TKIs were not available in China at that time. Now the third generation TKIs can be used, and therefore these patients will not be recruited into this trial.

## Disclosure

The authors declare that they have no competing interests.
